# Knockdown of Tripartite motif-containing 22 (TRIM22)relieved the apoptosis of lens epithelial cells by suppressing the expression of TNF receptor-associated factor 6 (TRAF6)

**DOI:** 10.1080/21655979.2021.1980645

**Published:** 2021-09-24

**Authors:** Kai Meng, Chengbo Fang

**Affiliations:** a Department of Ophthalmology, Fuyang Futian Eye Hospital, Fuyang, Anhui Province, China; b Department of Ophthalmology, The First Affiliated Hospital of Anhui Medical University, Hefei, Anhui Province, China

**Keywords:** Cataract, TRIM22, TRAF6, apoptosis, lens epithelial cells

## Abstract

Cataract is a disease that causes severe visual impairment in patients. Recent studies have found that lens epithelial cell apoptosis caused by oxidative damage is the critical cause of cataract. Moreover, TRIM22 could alleviate the ubiquitination of TRAF6. The expression of TRAF6 could activate the p38/MAPK pathway and aggravate the oxidative stress induced damage of lens epithelial cells. However, whether the TRIM22 could alleviate the oxidative stress induced damage of lens epithelial cells by regulating the expression of TRAF6 and p38/MAPK pathway is unclear. In this study, we stimulated the lens epithelial cells with the H_2_O_2_ and established the TRIM22 knockdown cells. Next, proliferation of these cells was determined by CCK-8 and EdU assays. Apoptosis of these cells was detected with the TUNEL assays. Levels of ROS was explored with the DCFH-DA staining. Finally, the expression levels of TRAF6, p-p38 and p-ERK were determined with the western blotting. According to the results, we found that knockdown of TRIM22 suppressed the proliferation and relieved the H_2_O_2_ induced DNA double-strand break and apoptosis of these cells. Inhibition of TRIM22 inhibited the production of ROS in these cells. Moreover, restriction of TRIM22 induced the decreased levels of TRAF6, p-p38 and p-ERK in lens epithelial cells. We concluded that inhibition of TRIM22 relieved the apoptosis of lens epithelial cells by suppressing the expression of TRAF6, p-p38 and p-ERK.

## Introduction

Lens opacity caused by cataract can induce dysopia and even blindness, which affect the living quality of patients [1]. There is no efficient path to prevent the formation of cataract lens, and surgical intervention can alleviate this symptom. However, surgery can cause recurrence of cataract, so cataract remains a major public health problem. Therefore, exploring the molecular mechanism of cataract is the crucial research direction. Recent study has found that the injury induced by the oxidative stress of the intraocular lens is the main cause of cataracts [2]. Hydrogen peroxide (H_2_O_2_) can induce oxidative stress and DNA double-strand breaks, which can cause DNA damage and apoptosis of lens epithelial cells [3,4]. This may also cause damage to the function of lens epithelial cells and tissues, and finally induce the cataract formation and cell death [5]. Therefore, how to prevent lens epithelial cells from being damaged by H_2_O_2_ is an urgent problem to be solved.

Tripartite motif-containing 22 (TRIM22) is a member of TRIM family of E3 ubiquitin ligase, also known as Staf50, located on chromosome 11, and contains an amino-terminal RING domain, a carboxy-terminal domain B30.2 domain and a B-box Structural domain [6]. TRIM22 played critical roles in regulating inflammation, apoptosis, and proliferation of multiple types of cells. Moreover, TRIM22 also promotes the expression of Bax and mediates the apoptosis of monocytes [7]. TRIM22 could activate NF-κB signaling and alleviate apoptosis of glioma cells by activating the degradation of IκBα [8]. In addition, inhibition of TRIM22 could relieve neuronal apoptosis and inflammation induced by hypoxia and glucose deprivation by repressing the NF-κB/NLRP3 axis [9]. Furthermore, through the oligonucleotide microarray hybridization experiment, results indicated that TRIM22 is highly expressed in the lens of cataract patients compared with the normal lens, which is statistically significant [10].

Study has revealed that TRIM22 could alleviate the self-ubiquitination of TNF receptor-associated factor 6 (TRAF6), and this effect was related to the RING domain of TRIM22 [11]. The overexpression of TRAF6 was associated to the development of oxidative stress and inflammation [12]. Inhibition of TRAF6 could also reduce the formation of choroidal neovascularization in the body, thereby inhibiting blindness in elderly patients [13]. In addition, the expression of TRAF6 can activate the MAPK pathway [14]. MAPK could regulate the lens epithelial cell damage caused by oxidative stress. And inhibition of p38-MAPK can alleviate oxidative stress induced apoptosis of lens epithelial cells [15,16]. In addition, activation of p38-MAPK could also induce DNA double-strand damage [17,18]. In summary, the above findings showed that p38-MAPK is the crucial pathway for regulating of oxidative stress damage.

In this study, we detected whether TRIM22 could modulate the human lens epithelial cells oxidative damage by regulating ubiquitination of TRAF6 and MAPK pathway. The results of our study could also provide the new therapy for the clinic treatment of cataract.

## Material and methods

### Cell culture and transfection

Human lens epithelial cell line (HLE-B3) was obtained from the ATCC (Manassas, VA, USA). These cells were cultured with RPMI-1640 medium supplemented with 10% Fetal Bovine Serum (Gibco, USA), and these cells were cultured in the incubator (37°C humid atmosphere, 5% CO_2_). TRIM22 knockdown (sequence: CTGAGAATTTAACTTTCGTTTCT) and TRAF6 overexpression lentivirus were obtained from the Genechem (Shanghai, China). Polybrene (Genechem, Shanghai, China) was applied for the promoting of transfection efficacy of these cells. Different concentration (0, 25, 50, 100 μm) of H_2_O_2_ was used for the stimulation (30 minutes) of these cells.

## Immunohistochemistry

20 lens epithelial tissue samples from the cataract patients with health bodies and 20 normal epithelial tissue samples were got from Fuyang Futian Eye Hospital. This assay was checked and approved by the ethics committee of Fuyang Futian Eye Hospital. These people were all aware of the contents of the experiments. Next, these tissues are embedded, sectioned and deparaffinized. Then, the tissues were incubated with H_2_O_2_ for the exposing of antigen sites. After that, BSA (Beyotime, China) was applied for block of the tissues. Then, the tissues were incubated with the primary antibodies at 4°C overnight. TRIM22 antibody (Abcam, ab224059) was used for this assay. Next, the tissues were incubated with the secondary antibody (Goat Anti-Rabbit IgG H&L, Abcam, ab205718) for 2 hours at room temperature. Then, the coloring agent (Thermo Fisher Scientific, USA) was added on the tissues. Finally, xylene was used for dehydrate of the tissue and the neutral gum was used for mounting [19]. The tissues were observed with the microscope (Lecia, Germany).

## CCK-8

Culture medium was used for the attenuation of CCK-8 (Dojindo, Japan). CCK-8 was incubated with these cells for 1 hour in the incubator. Next, the absorbance of these cells was explored with the spectrophotometer (Thermo Fisher Scientific, USA). The absorbance of the other cells was determined after 24, 48 and 72 hours.

## EdU assays

These cells were cultured in the 96 well plates. After the adhesion, these cells were incubated with the EdU (Beyotime, C0071S, China) for 1.5 hours. DAPI (Invitrogen, USA) was applied for the mark of the cell nucleus. Finally, the fluorescence of these cells was observed with the fluorescence microscope (Lecia, Germany).

## TUNEL staining

These cells were cultured in the 96 well plates. Then, the TUNEL (Beyotime, C1082, China) was incubated with these cells for 2 hours in the dark room. DAPI (Invitrogen, D21490, China) was applied for the mark of the cell nucleus. At last, fluorescence microscope (Lecia, Germany) was applied for the observation of the fluorescence of these cells.

## Immunofluorescence

These cells were plated in the 35 mm culture dish. Then, these cells were fixed with the 4% paraformaldehyde. Next, 0.5%Triton X-100 (Beyotime, China) was used for the membrane permeabilization of these cells. After that, primary antibody was incubated with these cells for 2 hours at room temperature. Primary antibody used in this research was γH2AX (Abcam, ab229914). Next, secondary antibody (Goat polyclonal Secondary Antibody to Rabbit IgG, Abcam, ab150077) was incubated with these cells in the dark room for 2 hours. Finally, DAPI (Invitrogen, USA) was used for the mark of cell nucleus and Laser Scanning Confocal Microscope (Olympus, Japan) was applied for the observation of these cells [20].

## Detaction of ROS

Cells were cultured in the six well plates. Next, DCFH-DA (Beyotime, S0033) was diluted with the serum-free medium and incubated with these cells in the incubator for 30 minutes. After that, these cells were washed with the PBS and the fluorescence was observed with the Laser Scanning Confocal Microscope.

## Detection of LDH

The supernatant of culture medium of these cells was collected with the sterile pipette. Commercial kits (Beyotime, C0016, China) was used for the detection of the levels of LDH in these cells. The operation of these assays was followed the instruction.

## ELISA assays

The medium supernatant of these cells was collected in the pipette. Human SOD ELISA kit (Abcam, ab171738), Human GSH-PX ELISA kit (Abcam, ab138881) and Human MDA ELISA kit (Abcam, ab17354) were used for the detection of the expression of SOD GSH-PX and MDA in these cells. All the operation was followed the instruction [21].

## RT-PCR

Trizol was used for the extraction of Total RNA in these cells. Next, RNA was reverse transcribed into cDNA with the commercial kits (Takara, Japan). After that, the ABI 7500 system (Thermo Fisher Scientific, USA) was applied for the amplification of cDNA. The 2^−∆∆Ct^ method was used for the analyzation of the results. The primers used in this research were TRIM22 forward primer 5ʹ-GAGGATCCCCGGGTACCGGTCG CCACCATGGATTTCTCAGTAAAGGTAGACATAG-3ʹ reverse primer 5ʹ-TCCTTGTAGTCCATACCGGAGCTCGGTGGG CACACAGTCATG-3ʹ GAPDH forward primer 5ʹ-TTCACCACCATGGAGAAGGC-3ʹ reverse primer 5ʹ-CCACCTGGTGCTCAGTGTAG-3ʹ [22].

## Western blotting

Lysis buffer (Beyotime, P0013 C, China) was used for the collection of protein samples. Next, the BCA was applied for the determination of the concentration of these samples. Then, these proteins were separated with the 10% SDS-PAGE gel (Beyotime, China). After that, the PVDF membranes (Millipore, USA) was used for the adsorption of proteins. The membranes were incubated with the primary antibodies at 4°C overnight. Primary antibodies used in this research were TRIM22 (Abcam, ab68071), Cleaved caspase-3 (Abcam, ab2302), Bax (Abcam, ab32503), Bcl-2 (Abcam, ab32124), TRAF6 (Abcam, ab33915), p-p38 (Abcam, ab170099), p38 (Abcam, ab31828), ERK (Abcam, ab32537), p-ERK (Abcam, ab192591) and β-actin (Abcam, ab8226). Next, the membranes were incubated with the secondary antibodies for 2 hours at room temperature. Finally, the bands were developed with the substrate (Millipore, USA). Image J was applied for the quantification of the bands [23].

## Statistical analysis

Graphpad Prism 6.0 was applied for the analyzation of the data in this research. Assays in this research were repeated for three times and the data in this research was displayed as mean ± SD. Student’s t test was used for the comparison between diverse groups. The difference was considered as statistic difference until the values of *p* was less than 0.05.

## Results

The proliferation and invasion of lens epithelial cells were associated with the occurrence of cataract. In this study, we speculated that the expression of TRIM22 could affect the oxidative stress and apoptosis of human lens epithelial cells by modulating the TRAF6, p-p38 and p-ERK. And we also detected the effect of TRIM22 on human lens epithelial cells in this research. The results of this study indicated that suppression of TRIM22 relieved the H_2_O_2_ induced oxidative stress and apoptosis of human lens epithelial cells. This conclusion could also provide the new strategy for the treatment of cataract.

## The expression of TRIM22 was enhanced in the cataract tissues

**Figure 1.**
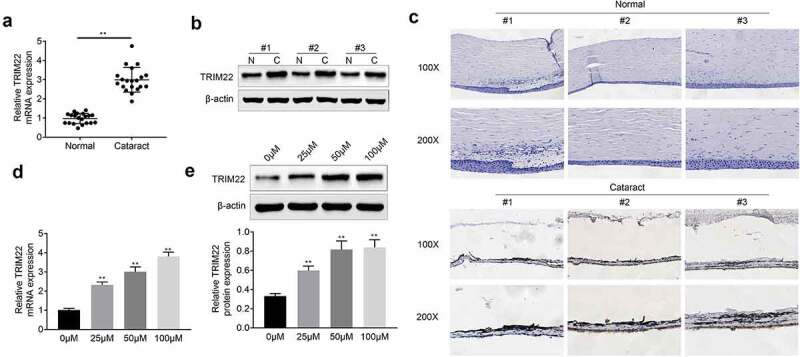
**The expression of TRIM22 was promoted in cataract tissues**. (a, b) The expression of TRIM22 in cataract tissues was detected with the RT-PCR and western blotting. (c) Immunohistochemical staining was performed for the detection of the expression of TRIM22 in cataract tissues. (d, e) The expression of TRIM22 in lens epithelial cells was detected with the RT-PCR and western blotting. ** *p* < 0.01 (Control *vs* H_2_O_2_)

For the detection of the effects of TRIM22 in the development of cataract, we explored the expression of TRIM22 in cataract tissues. According to the results (Figure 1(a) and Figure 1(b)), we found that mRNA and protein levels of TRIM22 were increased in the cataract tissues. As shown in Figure 1(c), results of immunohistochemical staining also showed that the expression of TRIM22 was promoted in the lens epithelial tissues of cataract patients. Next, H_2_O_2_ (0, 25, 50, 100 μm) was used for the stimulation of the human lens epithelial cells. The expression of TRIM22 in human lens epithelial cells was detected with the RT-PCR and western blotting. Results (Figure 1(d) and Figure 1(e)) showed that the expression of TRIM22 in these cells was enhanced after the stimulation with H_2_O_2_.

## Knockdown of TRIM22 recovered the proliferation of human lens epithelial cells

**Figure 2.**
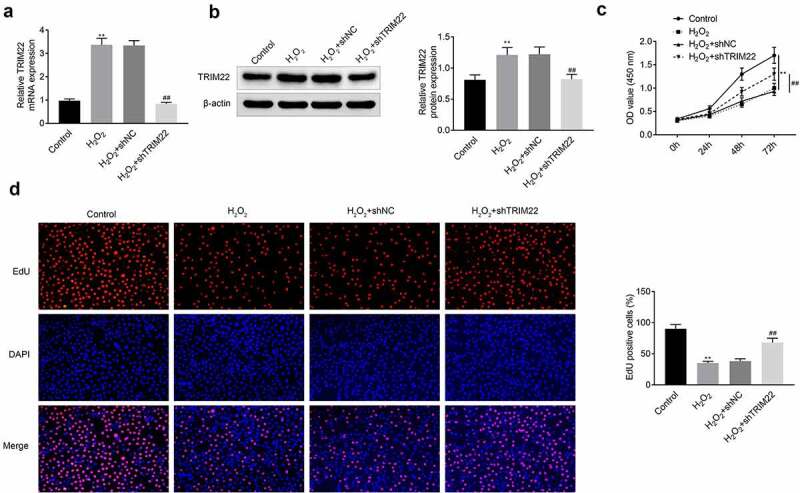
**Knockdown of TRIM22 suppressed the proliferation of lens epithelial cells**. (a, b) The expression of TRIM22 in lens epithelial cells was determined with the RT-PCR and western blotting. (c) CCK-8 was performed for the detection of the proliferation of lens epithelial cells. (d) EdU assays were performed for the detection of the proliferation of lens epithelial cells. ** *p* < 0.01, ## *p* < 0.05 (H_2_O_2_+ shNC *vs* H_2_O_2_+ TRIM22)

In this part, we established the TRIM22 knockdown human lens epithelial cells. Next, H_2_O_2_ (50 μm, 30 minutes) was applied for the stimulation of these cells. According to the results (Figure 2(a) and Figure 2(b)), the expression of TRIM22 was inhibited in TRIM22 knockdown human lens epithelial cells. Then, CCK-8 and EdU assays were performed for the detection of the proliferation of human lens epithelial cells. Results (Figure 2(c)) of CCK-8 showed that TRIM22 knockdown rescued the proliferation of human lens epithelial cells. Results (Figure 2(d)) of EdU assays also revealed that inhibition of TRIM22 promoted the proliferation of human lens epithelial cells.

## Knockdown of TRIM22 relieved the apoptosis of human lens epithelial cells

**Figure 3.**
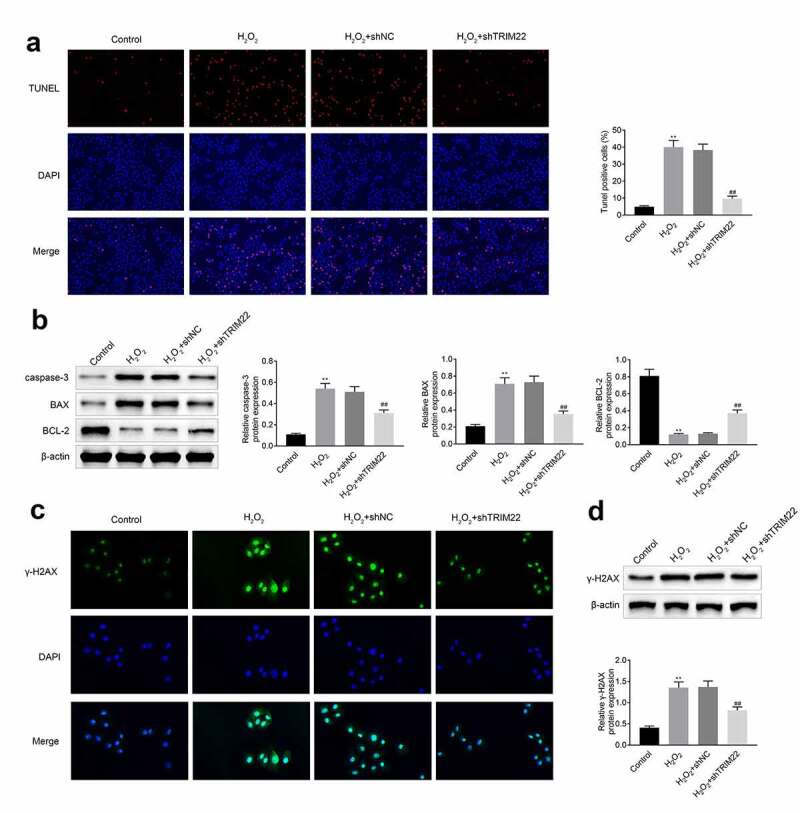
**Knockdown of TRIM22 relieved the apoptosis of lens epithelial cells**. (a) The apoptosis of lens epithelial cells was detected with the TUNEL staining. (b) The expression of apoptosis related proteins in lens epithelial cells was determined with the western blotting. (c, d) The expression of γ-H2AX in these cells was determined with the immunofluorescence and western blotting. ** *p* < 0.01, ## *p* < 0.05

We detected the apoptosis of human lens epithelial cells by TUNEL assays. As shown in Figure 3(a), H_2_O_2_ induced the apoptosis of human lens epithelial cells. Suppression of TRIM22 alleviated the H_2_O_2_ induced apoptosis of these cells. The expression of apoptosis related proteins Cleaved caspase 3 and Bax were decreased while the expression of Bcl-2 was enhanced in these cells (Figure 3(b)). DNA double-strand break was the reason of the apoptosis of cells. And the results of immunofluorescence and western blotting also showed that the expression of γ-H2AX was enhanced after the stimulation with H_2_O_2_. Moreover, the levels of γ-H2AX were decreased in human lens epithelial cells (Figure 3(c) and Figure 3(d)).

## Inhibition of TRIM22 relieved the oxidative stress of human lens epithelial cells

**Figure 4.**
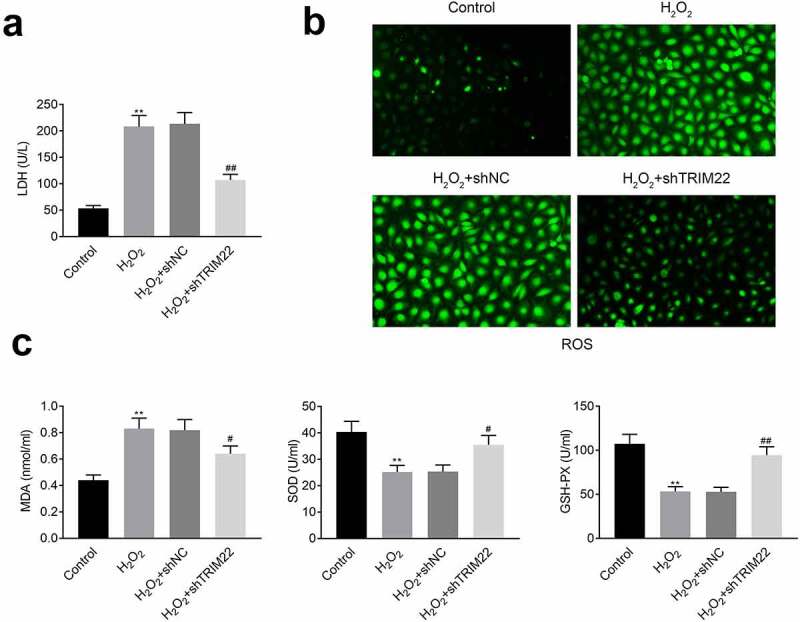
**Knockdown of TRIM22 relieved the oxidative stress of lens epithelial cells**. (a) The levels of LDH in lens epithelial cells was determined with the commercial kits. (b) The production of ROS was detected with the commercial kits. (c) The levels of MDA, SOD and GSH-PX were determined with the commercial kits. ** *p* < 0.01, ## *p* < 0.05

In this part, we detected the levels of LDH in these cells. Results (Figure 4(a)) showed that the levels of LDH were decreased after the inhibition of TRIM22. The production of ROS was also attenuated after knockdown of TRIM22 in human lens epithelial cells (Figure 4(b)). Next, ELISA assays were performed for the detection of the release of MDA, SOD and GSH-PX. According to the results (Figure 4(c)), stimulation with H_2_O_2_ promoted the production of MDA while suppressed the production of SOD and GSH-PX. Moreover, the levels of SOD and GSH-PX were rescued while the expression of MDA was restricted in the TRIM22 knockdown human lens epithelial cells.

## Knockdown of TRIM22 suppressed the p38 pathway by restricting the expression of TRAF6

**Figure 5.**
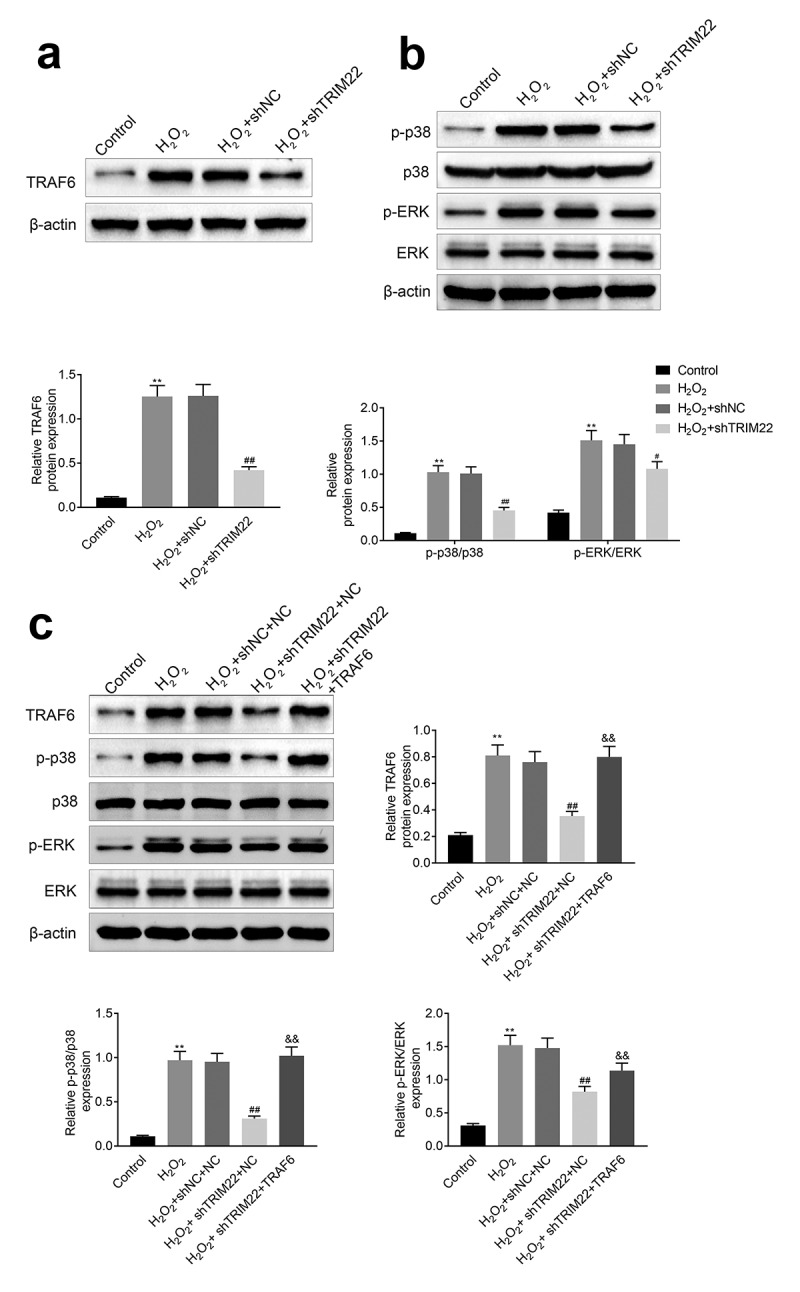
**Inhibition of TRIM22 suppressed the expression of TRAF6**. (a) The expression of TRAF6 in these cells were determined with the western blotting. (b) The expression of p-p38 and p-ERK in lens epithelial cells was determined with the western blotting. (c) The expression of TRAF6, p-p38 and p-ERK in lens epithelial cells was detected with the western blotting. ** *p* < 0.01, ## *p* < 0.05

In this part, we detected the expression of TRAF6, p-p38 and p-ERK in human lens epithelial cells. Results (Figure 5(a) and Figure 5(b)) showed that the expression levels of TRAF6, p-p38 and p-ERK were suppressed in TRIM22 knockdown human lens epithelial cells. Next, we overexpressed the TRAF6 in TRIM22 knockdown human lens epithelial cells and the expression of p-p38 and p-ERK was rescued in these cells (Figure 5(c)).

## Discussion

Lens opacity is a common complication of cataract surgery. 2–5 years after surgery, about 20%-40% of cataract patients have lens opacity-induced diminution of vision [24]. The occurrence of lens opacity is usually related to the proliferation, migration and epithelial-mesenchymal transition of the residual lens epithelial cells after surgery [25]. Therefore, it is very critical to explore the pathological changes of the lens during the onset of cataract. One study suggested that the oxidative damage induced the occurrence and development of cataract [26]. Oxidative stress induced DNA double-strand breaks in lens epithelial cells, which in turn induced cell apoptosis and impaired lens function [27]. Therefore, it is urgent to illuminate the molecular mechanism of the oxidative stress induced damage of lens epithelial cells.

In addition, TRIM22 as a ubiquitin ligase could participate in the regulation of cell inflammation and apoptosis [28]. Previous study indicated that the restriction of TRIM22 alleviated the ischemia reperfusion induced inflammation and apoptosis of neurocyte [9]. And the occurrence of enterogastritis was also associated with the expression of TRIM22 [29]. In this research, our results also suggested that the levels of TRIM22 were enhanced in the lens epithelial tissue of cataract sufferers. And the expression of TRIM22 was also promoted in lens epithelial cells after the stimulation of H_2_O_2_. The results also implied that the expression of TRIM22 activated the inflammation process during the development of cataract. Moreover, the proliferation of lens epithelial cells was associated with the occurrence and development of cataract [30]. There is also study revealed that the proliferation of lens epithelial cells could induce the occurrence and development of cataract [31]. We also revealed that the inhibition of TRIM22 rescued the proliferation of lens epithelial cells stimulated by H_2_O_2_.

Furthermore, apoptosis of lens epithelial cells caused by DNA double strand breaks was also the critical reason for the occurrence and development of cataract [32]. The signal of the occurrence of the DNA double strand breaks was the increased levels of γH2AX [33]. We also found that the expression of γH2AX was decreased and the apoptosis of lens epithelial cells was reduced after knockdown of TRIM22. On the other hand, the occurrence of oxidative stress could also lead to the apoptosis of lens epithelial cells and ultimately induce the occurrence of cataract [34]. As shown in the results, the production of ROS was also inhibited after the restriction of TRIM22. These results indicated that the inhibition of TRIM22 relieved the H_2_O_2_ induced apoptosis and oxidative stress of lens epithelial cells.

Moreover, TRAF6 is the member of the TRAF family and is considered as the signal converter of inflammatory signals [35,36]. Study has revealed that the expression of TRIM22 could induce the attenuation of the ubiquitination of TRAF6 [11]. The expression of TRAF6 could induce the activation of p38/MAPK pathway, therefore leading to the aggravation of apoptosis of lens epithelial cells [37]. In our research, we also revealed that the repression of TRIM22 could suppress the H_2_O_2_ induced increased levels of p-p38, p-ERK and TRAF6 in lens epithelial cells. Furthermore, the expression levels of p-p38 and p-ERK were recovered after the overexpression of TRAF6. These results suggested that TRIM22 activated the p38/MAPK pathway by promoting the expression of TRAF6 in these cells. The promotion of the expression of TRAF6 maybe due to the mitigatory ubiquitination of TRAF6, which needs to be further explored with the related assays. The results of this research implied that inhibition of TRIM22 alleviated the H_2_O_2_ induced apoptosis of human lens epithelial cells.

## Conclusion

Above all, we determined the effects of TRIM22 on the development of cataract. According to the results, we could conclude that knockdown of TRIM22 relieved the H_2_O_2_ induced oxidative stress and apoptosis of lens epithelial cells by activating the TRAF6 and p38/MAPK pathway. These findings our research could also provide the new strategy for the clinic treatment of cataract.

## Highlight

The expression of TRIM22 was enhanced in the cataract tissues

Knockdown of TRIM22 recovered the proliferation of human lens epithelial cells

Knockdown of TRIM22 relieved the apoptosis of human lens epithelial cells

Inhibition of TRIM22 relieved the oxidative stress of human lens epithelial cells

Knockdown of TRIM22 suppressed the p38 pathway by restricting the expression of TRAF6
